# Framework for determining the optimal course of action when efficiency and affordability measures differ by perspective in cost-effectiveness analysis—with an illustrative case of HIV treatment in Mozambique

**DOI:** 10.1186/s12962-023-00474-4

**Published:** 2023-09-13

**Authors:** Joseph Corlis, Jinyi Zhu, Hélder Macul, Orrin Tiberi, Makini A. S. Boothe, Stephen C. Resch

**Affiliations:** 1 Avenir Health, Takoma Park, MD USA; 2 Department of Health Policy, Vanderbilt University School of Medicine, Nashville, TN USA; 3 Programa Nacional de Controle de ITS-HIV/SIDA, Ministério da Saúde, Maputo, Mozambique; 4 UNAIDS, Maputo, Mozambique; 5 Center for Health Decision Science, Harvard T.H. Chan School of Public Health, Boston, MA USA

**Keywords:** Cost-effectiveness analysis, Patient perspective, Affordability, Value, Decision-making, HIV

## Abstract

**Background:**

Cost-effectiveness analysis (CEA) is a standard tool for evaluating health programs and informing decisions about resource allocation and prioritization. Most CEAs evaluating health interventions in low- and middle-income countries adopt a health sector perspective, accounting for resources funded by international donors and country governments, while often excluding out-of-pocket expenditures and time costs borne by program beneficiaries. Even when patients’ costs are included, a companion analysis focused on the patient perspective is rarely performed. We view this as a missed opportunity.

**Methods:**

We developed methods for assessing intervention affordability and evaluating whether optimal interventions from the health sector perspective also represent efficient and affordable options for patients. We mapped the five different patterns that a comparison of the perspective results can yield into a practical framework, and we provided guidance for researchers and decision-makers on how to use results from multiple perspectives. To illustrate the methodology, we conducted a CEA of six HIV treatment delivery models in Mozambique. We conducted a Monte Carlo microsimulation with probabilistic sensitivity analysis from both patient and health sector perspectives, generating incremental cost-effectiveness ratios for the treatment approaches. We also calculated annualized patient costs for the treatment approaches, comparing the costs with an affordability threshold. We then compared the cost-effectiveness and affordability results from the two perspectives using the framework we developed.

**Results:**

In this case, the two perspectives did not produce a shared optimal approach for HIV treatment at the willingness-to-pay threshold of 0.3 × Mozambique’s annual GDP per capita per DALY averted. However, the clinical 6-month antiretroviral drug distribution strategy, which is optimal from the health sector perspective, is efficient and affordable from the patient perspective. All treatment approaches, except clinical 1-month distributions of antiretroviral drugs which were standard before Covid-19, had an annual cost to patients less than the country’s annual average for out-of-pocket health expenditures.

**Conclusion:**

Including a patient perspective in CEAs and explicitly considering affordability offers decision-makers additional insights either by confirming that the optimal strategy from the health sector perspective is also efficient and affordable from the patient perspective or by identifying incongruencies in value or affordability that could affect patient participation.

**Supplementary Information:**

The online version contains supplementary material available at 10.1186/s12962-023-00474-4.

## Background

Bilateral and multilateral donors and foundations often conduct or commission economic evaluations to inform health program planning and to evaluate results [1–4]. Cost-effectiveness analyses (CEAs) represent one of the most widely known and used methodologies for economic evaluation, representing over 90% of published economic studies [5]. In 2016, the Second Panel on Cost-Effectiveness in Health and Medicine established the norm of reporting reference cases in CEAs using both societal and health sector perspectives [6]. Nevertheless, most CEAs of development assistance for health programs restrict their analysis to health sector costs—typically focusing on the donor and host-country government costs to provide health services and products [7]. Even studies that purport to adopt a societal perspective often exclude costs like patient travel time and lost wages [6, 8].

Patient costs can have a strong effect on participation [9, 10], which is a key factor in program effectiveness [11]. A common justification for excluding patient costs is that they are small relative to those of other payers (e.g., governments, donors) and do not substantially affect incremental cost-effectiveness ratios (ICERs) when included in analysis; however, even relatively small costs can impact patient behavior (i.e., intervention uptake, adherence) [12, 13].

Previous research shows that CEAs which complement a health sector or societal perspective with a distinct patient perspective provide vital information for health program decision-makers [14, 15]. The patient (sometimes called beneficiary) perspective considers the total out-of-pocket (OOP) expenses and time costs that an individual pays to access and use health products or services. Conducting a CEA with a companion patient perspective shows the value of interventions from a patient’s standpoint in the form of an ICER.

Donors such as the United States Agency for International Development already encourage their staff “to ‘seek out and respond to the priorities and perspectives of local stakeholders,’ including beneficiaries” [16]. Community-based participatory research, human-centered design, and similar methods have emerged as critical approaches for defining how development assistance for health programs are conceptualized, implemented, and evaluated [17, 18]. These methods can identify technical characteristics of health interventions that influence an individual’s propensity to participate, representing critical information for decision-makers aiming to improve intervention design and implementation. In that same spirit, CEAs represent an opportunity to consider the patient perspective of the economic characteristics of those health interventions.

Financial considerations are especially important in low- and middle-income countries (LMICs) where OOP costs often constitute a large share of total health spending [19, 20]. Even in instances that donors or governments fund health services and user fees have been eliminated (e.g., HIV programming), patients can still bear OOP expenses, time costs, and income losses that impact participation in health programs [21].

Our proposed methodology complements previous research regarding the systematic use of a patient perspective alongside the health sector or societal perspective [15], the affordability of cost-effective interventions [22, 23], and the ability for researchers to address situations when the optimal course of action varies by perspective in CEAs [14]. We suggest that researchers not only calculate ICERs from the patient and health sector perspectives but also determine the lump sum or annualized recurring cost for a patient to participate in the interventions. These costs can then be compared to an affordability threshold, such as the average annual OOP expenditures on health in a country or OOP health costs exceeding 10% of annual spending [24]. Finally, our methodology introduces a novel framework that researchers and policymakers can use to interpret the findings of the CEA, which can be applied regardless of the health outcome measure used in a given analysis. Critically, because a person’s ability to pay for health services can be measured in multiple ways [25], our methodology remains flexible for decision-makers to define an affordability threshold based on critical factors of the health issue in question (e.g., acute versus chronic condition, distribution among wealth quintiles).

Comparing the perspectives based on ICER and affordability calculations can reveal whether an intervention deemed cost-effective from the health sector perspective is also efficient and financially feasible for patients. This juxtaposition of the perspectives can equip health program planners with evidence that can potentially improve program design [7, 26].

Thus, the aim of this paper is three-fold: (1) to reinforce the importance of including a discrete patient perspective in CEAs for comparison with other perspectives, using the case of HIV treatment approaches in Mozambique; (2) to demonstrate a method for annualizing the costs of each intervention in a CEA to determine affordability; and (3) to provide practical guidance on how to compare the efficiency and affordability results that multiple CEA perspectives may produce. Our proposed methodology represents an improvement to current CEA methods by accounting for the financial feasibility of studied interventions—addressing a common critique of CEAs [27–30]—and by presenting a structure for using multiple perspectives in decision-making.

## Methods

### Modeling an example: the case of HIV treatment in Mozambique

To illustrate an application of the companion patient perspective, we analyzed six HIV treatment approaches in Mozambique from both the patient and health sector perspectives. More than 13% of Mozambique’s 32 million inhabitants live with HIV [31, 32]. International donors provide the majority of the country’s financial resources for HIV-related programs (97% of national expenditures in 2018), which total more than $500 million each year [33]. In 2021, it was estimated that, among people living with HIV (PLHIV) in Mozambique, 83% knew their status, while 74% of people who had been diagnosed with HIV underwent sustained antiretroviral therapy (ART), and 65% of people receiving ART achieved viral suppression [34].

Mozambique’s Fifth National Strategic Plan for HIV/AIDS Response [31] aims to reduce new HIV infections and AIDS-related deaths by 50% before 2025. However, loss to follow-up remains a major impediment to achieving the country’s viral suppression goals, with as much as 32% of PLHIV on ART registered as lost to follow-up (LTFU) 12 months after starting treatment [32, 35]. In light of the global SARS-CoV-2 outbreak, Mozambique’s Ministry of Health announced a nationwide shift in the standard of HIV care to clinical 3-month antiretroviral drug (ARV) distributions for patients who had been on ART for at least 3 months and whose HIV was not in clinical stages III or IV as defined by the World Health Organization [36, 37]. Prior to this 2020 shift, all PLHIV in the country received ART in clinical 1-month ARV distributions. In 2021, Mozambique began piloting clinical 6-month ARV distributions in 88 of the country’s 1709 health facilities that offer HIV care and treatment; these 88 facilities provide ART for 34% of all PLHIV on treatment in the country.

Although the country eliminated user fees for HIV-related services, other patient costs may further impact the individual’s perspective on the value for money of HIV treatment options. In 2020, 63.7% of Mozambique’s population lived below the international poverty line, and, as of 2018, the country’s average annual OOP expenditure for health was less than $4, below the low-income country average of $15 [38–40].

We used a Monte Carlo simulation with a 1-month cycle length for this study to include the pre-Covid-19 practice of 1-month ARV distributions. We populated the model with 200,000 individuals representing adult PLHIV (starting age, 15–80 years old) in the country. The time horizon of the study was 100 years with a lifetime analysis of cost-effectiveness and health outcomes. We measured health outcomes in terms of disability-adjusted life years (DALYs), a summary health outcome measure commonly used in low-income country settings, including Mozambique [41]. We conducted a probabilistic sensitivity analysis (PSA) using 1000 parameter sets for each perspective to incorporate parameter uncertainty in our model. Table 1 lists the model’s base-case values, sources, and sensitivity analysis ranges. Additional file 1 demonstrates the cost calculations used for the model’s interventions for each perspective. We conducted the modeling with TreeAge Pro Healthcare 2022, R1.2 software (Williamstown, Massachusetts), which used PERT distribution parameters to create beta distributions for the PSA [42].

**Table 1:** Parameter values used in the cost-effectiveness analysis of HIV treatment approaches in Mozambique

Parameter	Value(s)	Sensitivity analysis range	Probability distribution	Source(s)
Epidemiology				
Number of people $$\ge$$ 15 years old living with HIV	1,970,000 PLHIV	N/A	N/A	[47]
Age distribution of PLHIV $$\ge$$ 15 years old	15–19: 4.38% 20–24: 9.92% 25–29: 15.25% 30–34: 16.37% 35–39: 15.82% 40–44: 14.22% 45–49: 10.89% 50–54: 5.58% 55–59: 3.31% 60–64: 1.97% 65–69: 1.18% 70–74: 0.65% 75–80: 0.32% > 80: 0.15%	N/A	N/A	[47]
Distribution between CD4 count stages at time of ART initiation	CD4 > 500 cells/µL: 31% CD4 350–500 cells/µL: 19% CD4 200–349 cells/µL: 23% CD4 < 200 cells/µL: 27%	N/A	N/A	Internal INS data, 2022
Mortality rates per 100 person-years for individuals with untreated HIV, by CD4 stage (with 95% CI)	CD4 > 500 cells/µL: 0.6 CD4 350–500 cells/µL: 1.6 CD4 200–349 cells/µL: 4.2 CD4 < 200 cells/µL: 21.2	CD4 > 500 cells/µL: 0.1–2.0 CD4 350–500 cells/µL: 0.8–3.0 CD4 200–349 cells/µL: 2.8–5.7 CD4 < 200 cells/µL: 13.2–41.5	PERT distributions	[48]
CD4 count decrease per year without ART (range)	60 cells/µL	50–80 cells/µL	PERT distribution	[49]
CD4 count decrease per year while on ART (inadequate adherence, viral resistance)	14 cells/µL	0–20 cells/µL	PERT distribution	[50, 51]
CD4 count increase per year while on ART	205 cells/µL	170–250 cells/µL	PERT distribution	[52, 53]
12-month LTFU risk for 1-month clinical ARV distribution	20%	12–32%	PERT distribution	[32, 35]
LTFU rate for 3-month community ARV distribution	5.1 per 100 person-years	1.59–8.61 per 100 person-years	PERT distribution	[54]
Adjusted odds ratio of LTFU in 2 years with multi-month ARV distribution (compared to 1-month clinical distributions)	3-month: 0.79 6-month: 0.41	3-month: 0.76–0.82 6-month: 0.31–0.54	PERT distributions	[55]
Probability of reengaging in care 12 months after LTFU with/without patient tracing	With: 37.1% Without: 15.1%	With: 30–44% Without: 8–22%	PERT distribution	[56]
Proportion of PLHIV on ART taking first-line ARVs	95%	75–98%	PERT distribution	[57, 58]
Distribution of first-line ARV regimens by base-drug	Dolutegravir: 99% Efavirenz: 1%	N/A	N/A	Internal CNCS data, 2021
Risk of ART failing while on second-line ARVs	15 per 100 person-years	13–18 per 100 person-years	PERT distribution	[59]
Annual risk of progressing to second-line ARVs by base-drug regimen	Dolutegravir: 0.25% Efavirenz: 2.5%	Dolutegravir: 0–0.5% Efavirenz: 2.0–3.3%	PERT distributions	[57] [60, 61]
Discount rate	5%	N/A	N/A	[43]
Health effects				
DALY weight for one year while on ART, by CD4 stage	CD4 > 500 cells/µL: 0.078 CD4 350–500 cells/µL: 0.1 CD4 200–349 cells/µL: 0.15 CD4 < 200 cells/µL: 0.2	N/A	N/A	[62]
DALY weight for one year without ART, by CD4 stage	CD4 > 500 cells/µL: 0.012 CD4 350–500 cells/µL: 0.27 CD4 200–349 cells/µL: 0.377 CD4 < 200 cells/µL: 0.58	N/A	N/A	[62]
Costs (in 2020 US$ with adjustment for purchasing power parity in Mozambique for non-tradeable cost components)				
Currency conversion	US$1 = 73.37 meticais 1 metical = US$0.014	N/A	N/A	[63]
Annual cost to the health sector per patient treated for clinical 1-month ARV distributions (excluding ARV costs)	$134	$104–163	PERT distribution	[33]
Average annual cost to the health sector per patient treated by community 3-month ARV distributions (excluding ARV costs)	$160	$125–226	PERT distribution	[64]
Per person cost of first-line ARV regimens per month	Dolutegravir: $5.55 Efavirenz: $6.40	N/A	N/A	[65]
Per person cost of second-line ARV regimen per month	Ritonavir: $23.15	N/A	N/A	[65]
Annual cost to the health sector for viral load testing (per patient)	$26	$20–43	PERT distribution	[33]
Average monthly cost to the health sector for case management and patient support (per patient)	$37.97	$25–50	PERT distribution	[66, 67]
Per-person cost to travel by minibus taxi (*chapa*)	20 meticais (US$0.28) per hour	$0.05-$1.00	PERT distribution	[68]
Average round trip transit time to ART provision site (by *chapa*)	< 60 min: 51% 60–240 min: 43% > 240 min: 6%	N/A	N/A	[69]
Average round trip transit time to ART provision site (walking)	6 h	1–24 h	PERT distribution	[68, 70]
Proportion of patients who travel by *chapa* to ART services	57.5%	33–66%	PERT distribution	[71, 72]
Proportion of distance a patient travels to a mobile brigade ARV distribution site versus to a clinic	33%	15–50%	PERT distribution	Author estimation
Average patient wait time for ART	1 h	0.25–4 h	PERT distribution	[73]
OOP expenditure on health per capita in Mozambique	$4.14 per year	N/A	N/A	[39]
Adjusted net national income per capita in Mozambique	$393 per year	N/A	N/A	[74]
GDP per capita in Mozambique	$467 per year	N/A	N/A	[75]

All currency values are in 2020 US$

*ART* antiretroviral therapy, *ARV* antiretroviral drug, *CNCS* Mozambique’s National AIDS Council, *DALY* disability-adjusted life year, *GDP* gross domestic product, *HIV* human immunodeficiency virus, *INS* Mozambique’s National Health Institute, *LTFU* lost to follow-up, *OOP* out-of-pocket, *PLHIV* people living with HIV

**Fig. 1:**
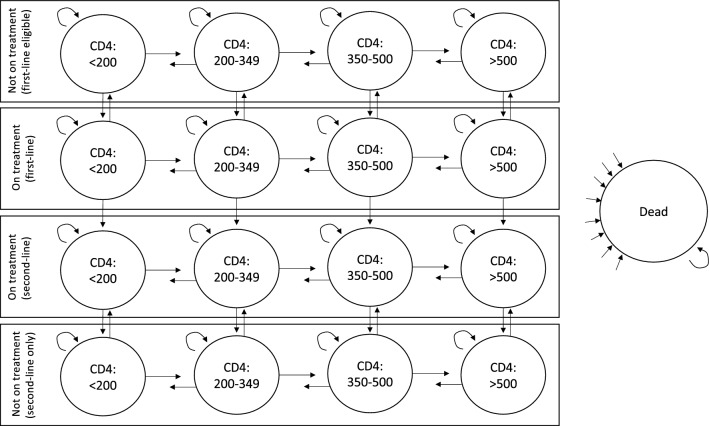
Diagram of the health states modeled. In this diagram, circles represent health states; rectangles represent treatment status; and arrows represent all possible transitions between health states. Each month, patients can remain in their previous health state, change to health states with different CD4 counts in the same treatment status, change to health states with the same CD4 counts in different treatment statuses, or die

Figure 1 illustrates 17 possible health states for HIV progression in the simulation. The model included different CD4 count states (< 200, 200–349, 350–500, and > 500) for four categories of people (PLHIV on first-line ARVs, PLHIV not on ART who remain eligible for first-line ARVs, PLHIV on second-line ARVs, and PLHIV not on ART who are not eligible for first-line ARVs). Each month, PLHIV could transition to higher or lower CD4 count states, remain in the same CD4 count state, transition to a different treatment status, or die. PLHIV could transition from first- to second-line ARVs only once. We half-cycle corrected all costs and health effects, and we discounted all health outcomes and costs at a 5% annual rate [43].

The CEA focused on a competing choice of individual treatment interventions for adults living with HIV. The six treatment interventions we considered were:

1. The pre-Covid-19 practice of distributing 1 month of ARVs at health facilities;
2. The current (status quo) practice of distributing 3 months of ARVs in health facilities;
3. Distributing 6 months of ARVs in health facilities;
4. Distributing 3 months of ARVs in health facilities paired with increased LTFU case management;
5. Distributing 3 months of ARVs in communities through mobile brigades; and
6. A mixed strategy of clinical 6-month ARV distribution for 50% of PLHIV on treatment and community 3-month ARV distribution via mobile brigades for the other 50% of PLHIV on treatment.

We modeled all interventions to follow the national standard of providing clinical 1-month ARV distributions to all patients with HIV in clinical stages III or IV as well as during the first 3 months of treatment. Harms considered in our modeling included time costs and adverse treatment reactions, but not stigma.

### Affordability analysis

For both perspectives, we converted the discounted total cost, *C*_T_, for each treatment approach into a stream of equivalent annual costs, *C*_*A*_, by using an annuity factor with a 5% discount rate, *d*, and a duration equal to the average number of years a patient spends on treatment for each intervention, *Y*_*T*_.

$${C}_{A}= {C}_{T} / \left(\frac{{1-(1+d)}^{{Y}_{T}}}{d}\right)$$

We then compared the annualized cost of the approach to an affordability threshold—in this case, the threshold was the average total patient OOP expenditure on health per year—to determine whether the intervention was affordable for patients.

### Comparing the perspectives

We compared the results of both perspectives to determine whether the CEA identified a common optimal strategy. Using a country-specific willingness-to-pay threshold of 0.3 × gross domestic product per capita (GDPpc) for each DALY averted [44–46], we determined the cost-effective treatments from the patient and health sector perspectives. If the two perspectives identified the same treatment approach as optimal, we confirmed whether the annual cost of the approach exceeded the study’s affordability threshold. In the absence of a common optimal approach for HIV treatment between the two perspectives, we first determined whether the optimal strategy from the health sector perspective was on the efficiency frontier from the patient perspective. We next checked whether the optimal strategy for the health sector perspective had an annual cost in the patient perspective that fell below the affordability threshold. Finally, we developed and followed the framework for comparing results (Table 2) to determine which recommendations to offer decision-makers. (For a more detailed version of Table 2 that includes further explanation of each consideration in the comparison process and examples, see Additional file 2.)

**Table 2:** Framework for comparing the results of multiple perspectives in CEAs

Result pattern	Efficiency results	Optimal intervention’s affordability for patients	Possible next steps in decision-making
Perfectly Congruent	The different perspectives identify the same intervention as optimal	Affordable	The CEA should recommend the optimal intervention
Weakly Congruent	The intervention which is optimal from the health sector perspective is also efficient from the patient perspective but not optimal at the willingness-to-pay threshold	Affordable	The CEA should recommend the intervention that is optimal from the health sector perspective. The CEA should also recommend that decision-makers redesign or implement the interventions in such a way that maximizes patient incentive to choose the health sector’s optimal intervention
Incongruent	The intervention which is optimal for the health sector is not efficient from the patient perspective	Affordable	
Consistent	The intervention which is optimal from the health sector perspective is also efficient from the patient perspective (may be optimal at the willingness-to-pay threshold or simply on the efficiency frontier)	Unaffordable	The CEA should recommend that decision-makers redesign the intervention which is optimal from the health sector perspective in order to decrease or offset the patient’s OOP expenditures, making the intervention more affordable for patients. If such a redesign does not generate an optimal intervention from the health sector perspective that is affordable to patients, then the intervention should be eliminated from consideration (but ICERs should not be recalculated) and the next most cost-effective intervention from the health sector perspective that is efficient and affordable from the patient perspective becomes optimal and should be recommended
Inconsistent	The intervention which is optimal for the health sector is not efficient for the patient perspective	Unaffordable	

*CEA* cost-effectiveness analysis, *ICER* incremental cost-effectiveness ratio, *OOP* out-of-pocket

## Results

### Value of HIV treatment approaches

Clinical 6-month ARV distributions are optimal from the health sector perspective and on the patient perspective’s efficiency frontier (Table 3). The status quo practice of clinical 3-month ARV distributions was dominated in the patient perspective, and the pre-Covid-19 practice of 1-month clinical ARV distributions was dominated in both perspectives.

**Table 3:** Cost-effectiveness of HIV treatment approaches in Mozambique (by perspective)

Intervention	Total cost per PLHIV	Total DALYs averted	Average survival	Average time on ART	Average annual cost on ART	Incremental cost^a^	Incremental DALYs averted^a^	ICER (cost per DALY averted)
Patient perspective								
Clinical 6-month ARV distribution	$24.15	10.79	24.6 years	16.9 years	$2.16	Reference	Reference	Reference
Mixed community 3-month and clinical 6-month ARV distribution	$25.29	11.21	25.9 years	19.2 years	$2.08	$1.14	0.42	$2.71
Community 3-month ARV distribution	$26.82	11.74	27.6 years	22.5 years	$2.01	$1.53	0.53	$2.89
Clinical 3-month ARV distribution (status quo)	$33.49	9.86	21.9 years	12.2 years	$3.72	$6.67	−1.88	Dominated
Clinical 3-month ARV distribution with LTFU case management	$47.03	11.92	28.5 years	20.7 years	$3.69	$20.21	0.18	$112.28
Clinical 1-month ARV distribution	$77.92	9.37	20.5 years	10.3 years	$9.83	$30.89	−2.55	Dominated
Health sector perspective								
Clinical 3-month ARV distribution (status quo)	$1284	9.87	21.9 years	12.2 years	$142	Reference	Reference	Reference
Clinical 6-month ARV distribution	$1405	10.80	24.6 years	16.8 years	$125	$121	0.93	$130
Clinical 1-month ARV distribution	$1548	9.39	20.6 years	10.4 years	$195	$143	−1.41	Dominated
Mixed community 3-month and clinical 6-month ARV distribution	$2073	11.22	25.9 years	19.2 years	$170	$668	0.42	$1590
Community 3-month ARV distribution	$2905	11.74	27.6 years	22.5 years	$218	$832	0.52	$1600
Clinical 3-month ARV distribution with LTFU case management	$6203	11.93	28.5 years	20.8 years	$486	$3298	0.19	$17,358

All values discounted 5% per year (except the average annual cost on ART), and all currency values are in 2020 USD. Slight differences in health outcomes for the same intervention were observed between the two different perspectives. This is due to first-order uncertainty in the model

*ART* antiretroviral therapy, *ARV* antiretroviral drug, *DALY* disability-adjusted life year, *HIV* human immunodeficiency virus, *ICER* incremental cost-effectiveness ratio, *LTFU* lost to follow-up, *PLHIV* person living with HIV

^a^Incremental costs and incremental DALYs averted are calculated as the increment between an intervention and the next non-dominated intervention

**Fig. 2:**
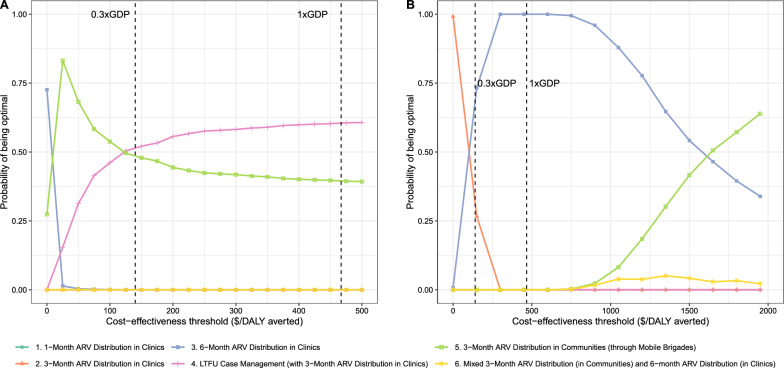
Cost-effectiveness acceptability curves for HIV treatment approaches in Mozambique from the **A** patient perspective and **B** health sector perspective. *ARV* antiretroviral drug, *DALY* disability-adjusted life year, *GDP* gross domestic product, *LTFU* lost to follow-up

We summarized the results of the PSA with the cost-effectiveness acceptability curves shown in Fig. 2. From the patient perspective (Fig. 2A), clinical 3-month ARV distribution with LTFU case management was optimal at 0.3 × GDPpc. The acceptability curves illustrated that the probability that another treatment approach was optimal decreased as the willingness-to-pay threshold increased. For example, clinical 6-month ARV distribution was cost-effective when the willingness to pay was less than $2.71 per DALY averted while community 3-month ARV distribution was cost-effective when the willingness to pay was between $2.89 and $112.28 per DALY averted. While the willingness-to-pay threshold for health gains likely varies widely among patients, it is reasonable to assume most patients in Mozambique would view $3 per DALY averted as very good value.

Figure 2B shows the cost-effectiveness acceptability curves for the analysis from the health sector perspective. Clinical 6-month ARV distribution was optimal at the 0.3 × GDPpc per DALY averted threshold. Other non-dominated approaches—e.g., community 3-month ARV distribution via mobile brigades, clinical 3-month ARV distribution with LTFU case management—exceeded 3 × GDPpc per DALY averted. Although donor-financed HIV programs may have higher willingness-to-pay thresholds than government-backed programs, the willingness to pay among bilateral and multilateral organizations is very unlikely to exceed 3 × GDPpc per DALY averted.

### Affordability of HIV treatment approaches

The average annual cost to PLHIV of the pre-Covid-19 practice of clinical 1-month ARV distributions may explain the country’s high LTFU rate, as $9.83 per person per year for ART is more than double (237%) the annual average for total OOP expenditures on health in Mozambique ($4.14). Meanwhile, community 3-month ARV distributions, which had the lowest annual cost from the patient perspective ($2.01 per patient per year of treatment), fell below the affordability threshold (49% of the average patient’s annual OOP expenditures on health).

The status quo treatment, which had the lowest lifetime cost per person treated for the health sector ($1284), had a higher annual cost per person on treatment from both perspectives (health sector: $142; patient: $3.72) than the clinical 6-month ARV distribution approach had (health sector: $125; patient: $2.16).

### Perspective alignment

Our model simulations revealed results with weak congruence between perspectives, meaning that the optimal strategy from the health sector perspective—clinical 6-month ARV distributions—was on the efficiency frontier and below the affordability threshold from the patient perspective. Thus, while clinical 6-month ARV distributions did not offer the highest value in both perspectives, the evidence supports scaling up this treatment approach for PLHIV throughout Mozambique. While we do not recommend discontinuing the offer of other differentiated treatment models for patients who may prefer other services for reasons not related to value for money, program planners should look for ways to incentivize patients to attend ART in clinics for 6-month ARV distributions whenever possible.

Importantly, because clinical 6-month ARV distributions cost the health sector perspective less annually than the pre-Covid-19 and status quo treatment approaches, implementing clinical 6-month distributions may also help the country bridge the resource needs gap demonstrated in the country’s National Strategic Plan for HIV/AIDS Response, 2021–2025 [31].

Furthermore, we note that while community 3-month ARV distributions offered very good value from the patient perspective, as well as the lowest annual cost to patients, the treatment approach’s ICER in the health sector perspective exceeded 3 × GDPpc per DALY averted. Thus, mobile brigades should not be widely rolled out in Mozambique without more evidence that either: the cost to the health sector of community 3-month ARV distributions can be reduced without sacrificing effectiveness, or the efficiency of mobile brigades can be improved by targeting patients who are at high risk of non-adherence within clinic-based ARV delivery.

## Discussion

Incorporating a patient perspective in a CEA can help uncover whether interventions, in particular those marketed to the public as “free”—as is often the case for HIV treatment in LMICs—still impose financial or opportunity costs that act as barriers to patient participation. By using our framework to account for multiple perspectives, health program planners can determine whether interventions which would represent good value for money for a key donor, the health sector, or society at large may, in fact, see the value of those interventions eroded due to low uptake by patients for whom cost is an obstacle. Considering multiple perspectives is especially critical in LMICs as domestic governments, private sector partners, and households assume a greater share of health financing and as previously siloed health services are integrated into more horizontal health programs [14]. Simply put, it is not enough to know which interventions are cost-effective from a health sector or societal perspective without knowing who bears those costs. Patient participation in health interventions can be highly sensitive to financial cost [12, 76], and the methodology we described and illustrated provides a check for patient value and affordability.

We do not suggest, however, that a patient’s perspective receive priority status over all other perspectives when making health program decisions. Instead, if the results of the different perspectives do not converge on the same optimal strategy (what we have termed a “perfectly congruent” comparison), researchers and program planners can use the results of the patient perspective to inform decision-making in several ways (Table 2).

In an *ex-ante* application of our methodology, decision-makers can select the intervention deemed optimal from the health sector perspective as long as the intervention does not exceed the affordability threshold for patients. When possible, we recommend that researchers use the results of *ex-ante* CEAs iteratively to reengineer the design of interventions in a way that makes the results perfectly congruent between perspectives (e.g., by adding a subsidy for travel costs to ART sites, by decreasing patient wait times for services, by reducing health system costs for LTFU patient tracking) or to create OOP cost scaffolds that drive patients toward the health sector perspective’s optimal strategy. In cases where the framework recommends redesigning the intervention to be more favorable from the patient perspective, revisions to the intervention may also affect the cost-effectiveness evaluated from the health sector perspective (e.g., making the intervention more favorable to patients could increase patient demand and generate improvements in efficiency for the health sector). When adjusting the costs of health interventions is not possible, we suggest that researchers use this methodology to highlight any interventions identified as unaffordable to patients (termed “consistent” or “inconsistent” in Table 2) so that health program planners can avoid recommending the implementation of those strategies.

In an *ex-post* application of the methodology, researchers and health program decision-makers can provide evidence that patient costs may have rendered an intervention unaffordable, potentially hindering participation. Conversely, researchers may determine that patient costs per year are so low that affordability likely does not limit participation in a given intervention. In such cases, decision-makers should use the ICER results and other metrics (e.g., equity, sustainability, other patient preferences) to prioritize future programming. Even in such instances, the methodology we propose serves as due diligence, equipping researchers and health program planners with critical information for discussions: with local communities about expected OOP expenditures, with donor organizations about the potential benefits of prioritizing specific interventions, and with LMIC governments about the costs of transitioning programs from donor portfolios to domestic budgets.

Our illustrative CEA of HIV treatment approaches in Mozambique had several limitations. First, our model, which we populated solely with PLHIV who already knew their status, did not account for the onward transmission of HIV across the general population as a dynamic or compartmental model would have [77, 78]. As a result, our model likely underestimated the health effects of each treatment approach by omitting the impact that effective HIV treatment can have on epidemic control. We note as well that our illustrative model—like many CEAs—focused on population-level averages. For researchers and policy-makers who are interested in variability among patient sub-populations or across clinical settings, our proposed framework can be applied to distributional cost-effectiveness analyses [79–81] or to CEAs that stratify patients or health service delivery settings in some other manner [82, 83]. In such models, researchers may see variations in the alignment of perspectives (e.g., weakly congruent, consistent) within sub-groups of patients and/or settings. Alternatively, a Monte Carlo simulation could assign individual patients with a level of wealth informed by population-level income distribution data, thereby allowing the researcher to calculate outcome measures such as “proportion of patients for whom the intervention is not affordable” even in cases when the intervention is affordable on average.

Second, the parameters for several model inputs (Table 1) were not based on data from Mozambique. Because some of the differentiated service delivery approaches included in the model only began during the Covid-19 pandemic—for example, community ARV distribution using mobile brigades which has only recently been implemented as a pilot in the country—country-specific evidence has not yet been generated to inform certain model inputs. Therefore, we approximated some values based on similar interventions in neighboring countries. We conducted a PSA to explore uncertainty in the model, and all parameters in our model using data from nearby countries included a sensitivity analysis range.

Third, we only considered LTFU case management as an add-on to the status quo treatment approach (clinical 3-month ARV distribution). It may be appropriate to model LTFU case management in conjunction with other ARV distribution types. However, as treatment adherence improves, the relative value of LTFU will decline. Lastly, the mixed treatment approach did not account for patient preferences between clinical and community ARV distributions. We assumed an even split of patients between the two interventions. Moreover, we did not modify the model parameters related to adherence in this approach. We expect adherence might improve when patients can select their preferred ARV distribution method, which would improve the cost-effectiveness profile for a mixed clinical-community strategy.

We also note that our methodology for annualizing health costs creates an average annual cost, which may mask fluctuations in costs from a given year to another. If an intervention has widely variable annual costs, it may be unaffordable to patients some of the time. In instances when patient costs fluctuate substantially over time, researchers can either: (1) base the affordability threshold determination on the year with the highest patient costs (if known) or (2) use alternative methods for modeling and calculating annual patient costs to determine and account for variability. Our proposed framework for comparing the results of patient and health sector perspectives can accommodate either solution.

## Conclusions

This study produced a methodology for juxtaposing CEA results from the patient and health sector perspectives as well as for analyzing patient costs for interventions in a way that may help decision-makers identify optimal health program strategies. In the case of HIV in Mozambique, an exploration of the impact that differentiated service delivery models, which were rolled out at an accelerated pace due to the Covid-19 pandemic, have had on patient retention in care may support the findings of this analysis or provide more accurate data for future modeling using the same methodology.

Beyond Mozambique, this methodology should be replicated in other contexts and for other health areas—particularly for health programs that continue to impose opportunity costs or user fees on patients—to determine its suitability for widespread use among bilateral and multilateral donors, private foundations, LMIC governments, researchers, and other global health professionals.

### Supplementary Information

**Additional file 1: **Model cost calculations for HIV treatment approaches in Mozambique

**Additional file 2: **Expanded explanation of our framework for addressing situations where the optimal course of action in a cost-effectiveness analysis varies by perspective

## Data Availability

The datasets used and/or analyzed during the current study are available from the corresponding author on reasonable request.

## Abbreviations

AIDS: Acquired immune deficiency syndrome
ART: Antiretroviral therapy
ARV: Antiretroviral drug
CEA: Cost-effectiveness analysis
CNCS: Mozambique’s National AIDS Council
Covid-19: Illness caused by SARS-CoV-2
DALY: Disability-adjusted life year
GDP: Gross domestic product
GDPpc: Gross domestic product per capita
HIV: Human immunodeficiency virus
ICER: Incremental cost-effectiveness ratio
INS: Mozambique’s National Health Institute
LMIC: Low- and middle-income country
LTFU: Lost to follow-up
PLHIV: People living with HIV
PSA: Probabilistic sensitivity analysis
OOP: Out-of-pocket
SARS-CoV-2: Severe acute respiratory syndrome coronavirus 2
