# Supplementary material for: Framework for determining the optimal course of action when efficiency and affordability measures differ by perspective in cost-effectiveness analysis—with an illustrative case of HIV treatment in Mozambique
Source: Cost Eff Resour Alloc. 2023 Sep 13;21:62. doi: 10.1186/s12962-023-00474-4 (PMC10498553; doi:10.1186/s12962-023-00474-4)
Supplement: Supplementary file 1 — Additional file 1: Model cost calculations for HIV treatment approaches in Mozambique [file 12962_2023_474_MOESM1_ESM.docx]

**Supplemental Document 1: Model cost calculations for HIV treatment approaches in Mozambique**

*Part 1: Patient perspective cost variables:*

W_S_ = distribution of patient wait times (in hours) for ART services

T_T_ = distribution of patient travel times (in hours) by taxi

T_W_ = distribution of patient travel times (in hours) walking

T_C_ = proportion of distance a patient travels to mobile brigade ARV distribution sites versus to clinics

C_M_ = distribution of the hourly cost to miss work

C_S_ = distribution of the cost for taxis

P_T_ = probability that a patient travels to ART by taxi

P_W_ = probability that a patient walks to ART

*Part 2: Patient perspective cost calculations:*

Clinical 1-month ARV distributions:

= [W_S_ * C_M_] + [P_T_ * T_T_ * (C_S_ + C_M_)] + [P_W_ * T_W_ * C_M_]

= [costs to attend ART] + [costs for taxi travel] + [costs for walking]

Clinical 3-month ARV distributions:

= ([W_S_ * C_M_] + [P_T_ * T_T_ * (C_S_ + C_M_)] + [P_W_ * T_W_ * C_M_]) / 3

= ([costs to attend ART] + [costs for taxi travel] + [costs for walking]) / [once every 3 months]

Clinical 6-month ARV distributions:

= ([W_S_ * C_M_] + [P_T_ * T_T_ * (C_S_ + C_M_)] + [P_W_ * T_W_ * C_M_]) / 6

= ([costs to attend ART] + [costs for taxi travel] + [costs for walking]) / [once every 6 months]

Clinical 3-month ARV distributions with LTFU case management:

= ([W_S_ * C_M_] + [P_T_ * T_T_ * (C_S_ + C_M_)] + [P_W_ * T_W_ * C_M_]) / 3

= ([costs to attend ART] + [costs for taxi travel] + [costs for walking]) / [once every 3 months]

Community 3-month ARV distributions via mobile brigades:

= ([W_S_ * C_M_] + [P_T_ * (T_T_ * T_C_) * (C_S_ + C_M_)] + [P_W_ * (T_W_ * T_C_) * C_M_]) / 3

= ([costs to attend ART] + [costs for taxi travel] + [costs for walking]) / [once every 3 months]

Mixed clinical 6-month ARV distributions and community 3-month ARV distributions:

= (clinical 6-month costs * 0.5) + (community 3-month costs * 0.5)

*Part 3: Health sector perspective cost variables*

C_B_ = distribution of operational costs for mobile brigades

C_P_ = distribution of operational costs to provide ARVs

C_L_ = distribution of the costs for laboratory testing

C_C_ = distribution of the costs for case management

C_E_ = Cost of Efavirenz

C_V_ = Cost of Dolutegravir

C_R_ = Cost of Ritonavir

P_E_ = probability that a patient takes Efavirenz

P_V_ = probability that a patient takes Dolutegravir

*Part 4: Health sector perspective cost calculations*

Clinical 1-month distributions of first-line ARVs:

= C_L_ + [(P_E_ * C_E_) + (P_V_ * C_V_)] + C_P_

= [costs for laboratory testing] + [costs for antiretroviral drugs] + [costs for clinical operations]

Clinical 1-month distributions of second-line ARVs:

= C_L_ + C_R_ + C_P_

= [costs for laboratory testing] + [costs for antiretroviral drugs] + [costs for clinical operations]

Clinical 3-month distributions of first-line ARVs:

= C_L_ + [(P_E_ * C_E_) + (P_V_ * C_V_)] + [C_P_ / 3]

= [costs for laboratory testing] + [costs for antiretroviral drugs] + [costs for clinical operations / every 3 months]

Clinical 3-month distributions of second-line ARVs:

= C_L_ + C_R_ + [C_P_ / 3]

= [costs for laboratory testing] + [costs for antiretroviral drugs] + [costs for clinical operations / every 3 months]

Clinical 6-month distributions of first-line ARVs:

= C_L_ + [(P_E_ * C_E_) + (P_V_ * C_V_)] + [C_P_ / 6]

= [costs for laboratory testing] + [costs for antiretroviral drugs] + [costs for clinical operations / every 6 months]

Clinical 6-month distributions of second-line ARVs:

= C_L_ + C_R_ + [C_P_ / 6]

= [costs for laboratory testing] + [costs for antiretroviral drugs] + [costs for clinical operations / every 6 months]

Clinical 3-month distributions of first-line ARVs with LTFU case management:

= C_L_ + [(P_E_ * C_E_) + (P_V_ * C_V_)] + [(C_P_ + C_C_) / 3]

= [costs for laboratory testing] + [costs for antiretroviral drugs] + [(costs for operations & case management) / every 3 months]

Clinical 3-month distributions of second-line ARVs with LTFU case management:

= C_L_ + C_R_ + [(C_P_ + C_C_) / 3]

= [costs for laboratory testing] + [costs for antiretroviral drugs] + [(costs for operations & case management) / every 3 months]

Community 3-month distributions of first-line ARVs via mobile brigades:

= C_L_ + [(P_E_ * C_E_) + (P_V_ * C_V_)] + [C_B_ / 3]

= [costs for laboratory testing] + [costs for antiretroviral drugs] + [costs for mobile brigade operations / every 3 months]

Community 3-month distributions of second-line ARVs via mobile brigades:

= C_L_ + C_R_ + [C_B_ / 3]

= [costs for laboratory testing] + [costs for antiretroviral drugs] + [costs for mobile brigade operations / every 3 months]

Mixed clinical 6-month distributions and community 3-month distributions of first-line ARVs:

= (clinical 6-month distribution costs for first-line ARVs * 0.5) + (community 3-month distribution costs for first-line ARVs * 0.5)

Mixed clinical 6-month distributions and community 3-month distributions of second-line ARVs:

= (clinical 6-month distribution costs for second-line ARVs * 0.5) + (community 3-month distribution costs for second-line ARVs * 0.5)
