# Supplementary material for: Framework for determining the optimal course of action when efficiency and affordability measures differ by perspective in cost-effectiveness analysis—with an illustrative case of HIV treatment in Mozambique
Source: Cost Eff Resour Alloc. 2023 Sep 13;21:62. doi: 10.1186/s12962-023-00474-4 (PMC10498553; doi:10.1186/s12962-023-00474-4)
Supplement: Supplementary file 2 — Additional file 2: Expanded explanation of our framework for addressing situations where the optimal course of action in a cost-effectiveness analysis varies by perspective [file 12962_2023_474_MOESM2_ESM.docx]

**Supplemental Document 2: Expanded explanation of our framework for addressing situations where the optimal course of action in a cost-effectiveness analysis varies by perspective**

The framework we present describes five possible result patterns for comparing results of multiple perspectives conducted within the same cost-effectiveness analysis (CEA). We developed this framework to assist researchers and program planners in considering CEA results from multiple perspectives. Our framework relies on findings related to the efficiency of the health interventions across the different perspectives used in the CEA as well as the affordability of the interventions for patients. The framework identifies five possible result patterns: perfectly congruent, weakly congruent, incongruent, consistent, and inconsistent.

We use several terms in this methodology which bear defining, such as:

- *Optimal* – the intervention that produces the greatest benefit (e.g., most DALYs averted) without its incremental cost-effectiveness ratio exceeding the willingness-to-pay threshold;
- *Efficient* – any intervention that is on the efficiency frontier; and
- *Affordable* – any intervention that does not exceed the affordability threshold.

General first steps for using this framework include identifying two sets of information: (1) setting a willingness-to-pay (WTP) threshold for the analysis and determining the efficiency results of the interventions from each perspective, and (2) setting an affordability threshold for patient costs—which can be based on average annual out-of-pocket health spending, GDP, GNI, or some other measure—and then determining which of the health interventions are affordable from the patient perspective. Equipped with these two pieces of information, a researcher can use the framework described in this article.

**Table A** Result patterns in our methodology for using efficiency and affordability results from multiple perspectives in a cost-effectiveness analysis

| **Result Pattern** | **Intervention Efficiency** | **Affordability for Patients** |
| --- | --- | --- |
| Perfectly congruent | Same intervention is optimal from health sector and patient perspectives | Affordable |
| Weakly congruent | Intervention from health sector perspective is efficient (but not optimal) for patients | Affordable |
| Incongruent | Intervention from health sector perspective is not efficient for patients | Affordable |
| Consistent | Intervention from health sector perspective is efficient or optimal for patients | Unaffordable |
| Inconsistent | Intervention from health sector perspective is not efficient for patients | Unaffordable |

Each of these result patterns is described below in greater detail and with examples.

**Result Pattern 1: Perfectly Congruent**

Under this pattern, the health sector/societal perspective and the patient perspective both identify the same intervention as optimal. This means that while other interventions may be on the efficiency frontier for one or both of the perspectives, the perspectives both identify the same intervention as cost-effective at the set WTP threshold. As well, the optimal intervention falls below the affordability threshold for patient costs, meaning that the intervention is the cost-effective choice from the patient perspective, the cost-effective choice from the health sector or societal perspective, and affordable for patients.

**Table B** Perfectly Congruent Example

| **Intervention** | **Total cost** | **Total DALYs averted** | **Average annual cost** | **Incremental cost*** | **Incremental DALYs averted*** | **ICER (cost per DALY averted)** |
| --- | --- | --- | --- | --- | --- | --- |
| *Patient Perspective* | | | | | | |
| Intervention A | $50 | 5 | $8 | reference | reference | reference |
| Intervention B | $200 | 20 | $12 | $150 | 15 | $10 |
| Intervention C | $400 | 14 | $16 | $200 | -6 | dominated |
| *Health Sector Perspective* | | | | | | |
| Intervention A | $300 | 5 | $145 | reference | reference | reference |
| Intervention C | $1100 | 14 | $260 | $800 | 9 | $89 |
| Intervention B | $1700 | 20 | $305 | $600 | 6 | $100 |

In Table B, Intervention B is the cost-effective choice from both the health sector and patient perspectives at a WTP threshold of $100. Given that Intervention B is also below the affordability threshold of $15 for the Average Annual Cost for patients, this intervention is considered affordable. Under this methodology, these perspectives are considered perfectly congruent and the CEA provides evidence that health program planners should select Intervention B.

**Result Pattern 2: Weakly Congruent**

Under this pattern, the health sector or societal perspective identifies one intervention as optimally efficient at the set WTP threshold, but a different intervention is optimal from the patient perspective. Nevertheless, the optimal intervention from the health sector/societal perspective is efficient from the patient perspective (i.e., on the efficiency curve). As well, in this result pattern, the intervention which is optimal from the health sector or societal perspective falls below the affordability threshold for patient costs.

**Table C** Weakly Congruent Example

| **Intervention** | **Total cost** | **Total DALYs averted** | **Average annual cost** | **Incremental cost*** | **Incremental DALYs averted*** | **ICER (cost per DALY averted)** |
| --- | --- | --- | --- | --- | --- | --- |
| *Patient Perspective* | | | | | | |
| Intervention A | $50 | 8 | $13 | reference | reference | reference |
| Intervention B | $200 | 10 | $15 | $150 | 2 | $75 |
| Intervention C | $450 | 7 | $19 | $250 | -3 | $125 |
| *Health Sector Perspective* | | | | | | |
| Intervention C | $3000 | 7 | $240 | reference | reference | reference |
| Intervention A | $3100 | 8 | $258 | $100 | 1 | $100 |
| Intervention B | $3900 | 10 | $321 | $900 | 2 | dominated |

Using the example of Table C, Intervention B is optimal from the patient perspective at the $100 WTP threshold, but Intervention A is optimal from the health sector perspective. Because Intervention A is on the efficiency curve from the patient perspective and it is below the affordability threshold of $15 for the Average Annual Cost for patients, the perspectives are said to be weakly congruent and the CEA provides evidence that health program planners should select Intervention A.

Additionally, this CEA example should recommend that health policymakers and program planners adjust the design of Intervention A so that it is also optimal from the patient perspective and patients have incentive to participate (e.g., providing vouchers, modifying service hours).

**Result Pattern 3: Incongruent**

In cases where the health sector or societal perspective identifies an intervention as cost-effective which is affordable for patients but is not on the efficiency curve for the patient perspective, the perspectives are said to be incongruent. This could be because the intervention is dominated or exceeds the WTP threshold from the patient perspective. Table D demonstrates this result pattern.

**Table D** Incongruent Example

| **Intervention** | **Total cost** | **Total DALYs averted** | **Average annual cost** | **Incremental cost*** | **Incremental DALYs averted*** | **ICER (cost per DALY averted)** |
| --- | --- | --- | --- | --- | --- | --- |
| *Patient Perspective* | | | | | | |
| Intervention C | $50 | 2 | $7 | reference | reference | reference |
| Intervention A | $110 | 6 | $13 | $60 | 4 | $15 |
| Intervention B | $120 | 5 | $14 | $10 | -1 | dominated |
| *Health Sector Perspective* | | | | | | |
| Intervention C | $1800 | 2 | $240 | reference | reference | reference |
| Intervention B | $2042 | 5 | $258 | $242 | 3 | $81 |
| Intervention A | $2900 | 6 | $321 | $858 | 1 | $858 |

In Table D, Intervention B is optimal from the health sector perspective at the WTP threshold of $100 but dominated from the patient perspective, which means that it is not only not optimal but not on the efficiency curve from the patient perspective. Intervention B is below the $15 affordability threshold for average annual patient cost.

Under this methodology, this example is classified as incongruent. While the CEA does provide evidence that health program planners should implement Intervention B, the CEA should include strong recommendations that the decision-makers redesign Intervention B in such a way that it maximizes patient incentives to choose the intervention.

**Result Pattern 4: Consistent**

Under this result pattern, the intervention which is optimal from the health sector perspective is also efficient from the patient perspective (i.e., the intervention may be optimal at the WTP threshold or simply on the efficiency frontier). This intervention, however, is not affordable for patients because it exceeds the affordability threshold. Table E below illustrates this result pattern.

**Table E** Consistent Example

| **Intervention** | **Total cost** | **Total DALYs averted** | **Average annual cost** | **Incremental cost*** | **Incremental DALYs averted*** | **ICER (cost per DALY averted)** |
| --- | --- | --- | --- | --- | --- | --- |
| *Patient Perspective* | | | | | | |
| Intervention A | $50 | 5 | $15 | reference | reference | reference |
| Intervention B | $200 | 20 | $19 | $150 | 15 | $10 |
| Intervention C | $400 | 14 | $22 | $200 | -6 | dominated |
| *Health Sector Perspective* | | | | | | |
| Intervention A | $300 | 5 | $145 | reference | reference | reference |
| Intervention C | $1100 | 14 | $260 | $800 | 9 | $89 |
| Intervention B | $1700 | 20 | $305 | $600 | 6 | $100 |

In Table E, Intervention B is optimal from both the health sector and patient perspectives at the $100 WTP threshold. The Average Annual Cost to patients for this intervention, however, exceeds the $15 affordability threshold.

In this example, the perspectives are considered consistent, which means that although the CEA provides evidence that the same intervention is cost-effective from both perspectives, the CEA also provides evidence that patients are unlikely to be able to afford the cost-effective intervention.

Therefore, the CEA should recommend that decision-makers redesign the intervention which is optimal from the health sector perspective in order to decrease or offset the patient’s out-of-pocket expenditures, making the intervention more affordable for patients. If such a redesign does not generate an optimal intervention from the health sector perspective that is affordable to patients, then the researcher(s) should eliminate that intervention from consideration in the CEA’s analysis (but ICERs should not be recalculated) and the next most cost-effective intervention from the health sector perspective that is efficient and affordable from the patient perspective should be recommended. Using the example given in Table E, either Intervention B should be redesigned in a way that lowers the average annual patient cost from $19 to $15 or less, or the CEA should note that Intervention A, which is on the efficiency frontier from both perspectives and is affordable for patients, is recommended because of Intervention B’s high patient cost.

**Result Pattern 5: Inconsistent**

If perspectives have inconsistent results, the intervention which is optimal from the health sector or societal perspective is neither efficient nor affordable from the patient perspective.

**Table F** Inconsistent Example

| **Intervention** | **Total cost** | **Total DALYs averted** | **Average annual cost** | **Incremental cost*** | **Incremental DALYs averted*** | **ICER (cost per DALY averted)** |
| --- | --- | --- | --- | --- | --- | --- |
| *Patient Perspective* | | | | | | |
| Intervention C | $50 | 2 | $27 | reference | reference | reference |
| Intervention A | $110 | 6 | $23 | $60 | 4 | $15 |
| Intervention B | $120 | 5 | $24 | $10 | -1 | dominated |
| *Health Sector Perspective* | | | | | | |
| Intervention C | $1800 | 2 | $240 | reference | reference | reference |
| Intervention B | $2042 | 5 | $258 | $242 | 3 | $81 |
| Intervention A | $2900 | 6 | $321 | $858 | 1 | $858 |

In Table F, Intervention B is optimal from the health sector perspective at the $100 WTP threshold but it is dominated from the patient perspective. As well, the Average Annual Cost to patients for Intervention B exceeds the $15 affordability threshold. Therefore, Intervention B is neither efficient nor affordable from the patient perspective.

In this example, the perspectives are considered inconsistent. Such a CEA provides evidence that the perspectives do not share an optimal course of action. Health program planners should use this information to redesign the interventions (and potentially rerun the analysis) to improve the efficiency and affordability of the interventions from the patient perspective in order to generate more overlap between the perspectives.

Alternatively, program planners can prioritize the health sector perspective over the patient perspective. In such case, we recommend that, at the very least, decision-makers and health program implementers decrease or offset the patient’s out-of-pocket expenditures, reducing the financial burden that the intervention has on patients.
